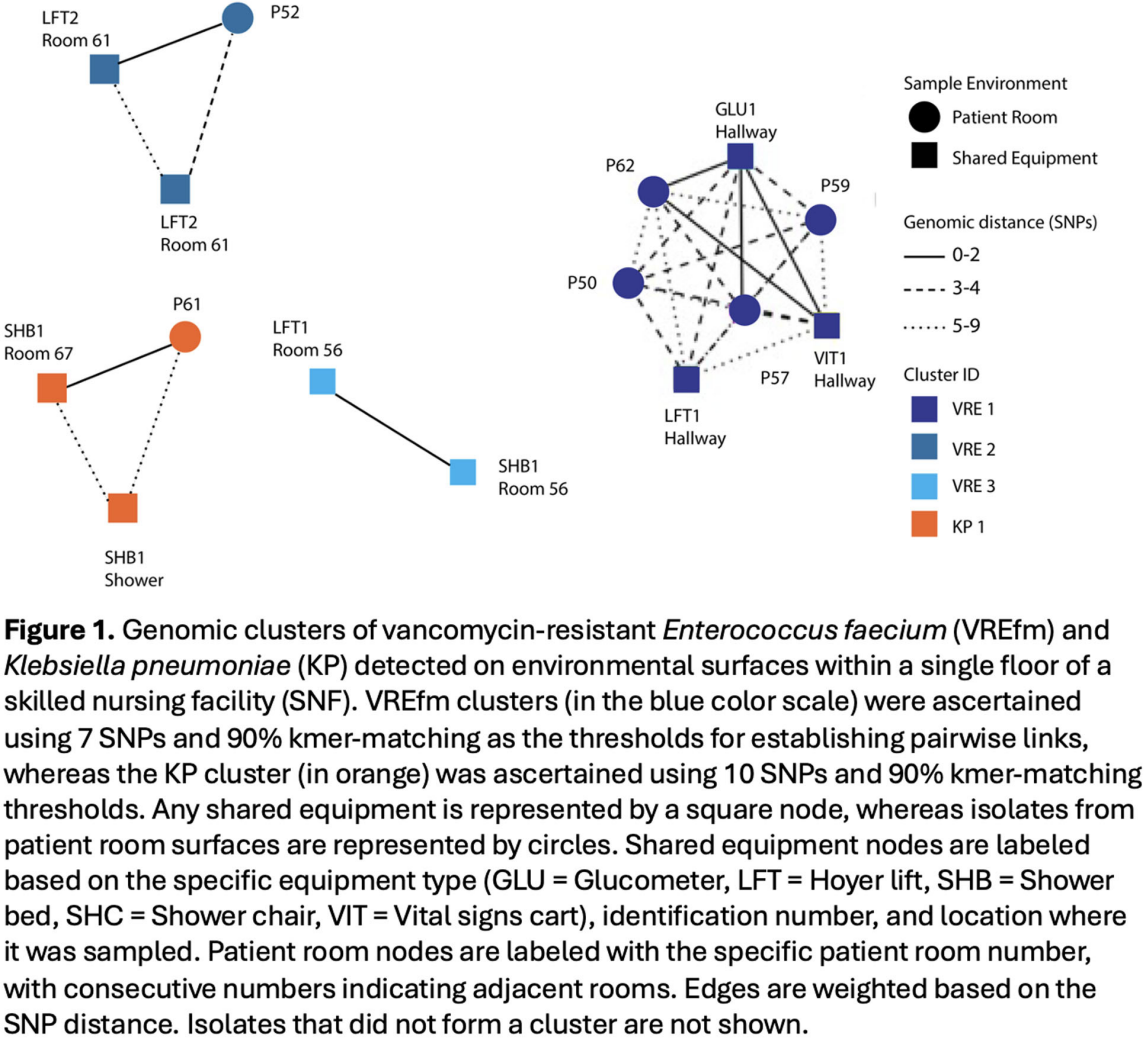# 154 ‘Can’tida auris’: When Candida auris can’t be controlled

**DOI:** 10.1017/ash.2026.10722

**Published:** 2026-06-23

**Authors:** Tierney O’Sullivan, Lindsay Keegan, Karim Khader, Lindsay Visnovsky, Frank Drews, Matthew Samore, Dorsan Egenia, Tavis Huber, Kristina Stratford

**Affiliations:** 1 University of Utah; 2 University of Utah School of Medicine; 3 Veterans affairs/University of Utah; 4 VA Salt Lake City Health Care System, University of Utah School of Medicine

## Abstract

**Background:** Multidrug-resistant organisms (MDROs) are prevalent in skilled nursing facilities (SNFs), but patterns of pathogen movement in these facilities are not well understood. We used genomic surveillance to identify MDRO presence on environmental surfaces, focusing on shared mobile medical equipment (MME) and patient room surfaces, to assess contamination patterns in a skilled nursing facility setting. **Methods:** We conducted environmental microbiological sampling in an 18-bed floor of a community-based, ventilator-capable SNF over four consecutive days. We collected composite samples of patient room environments and five types of shared MME: glucometers, vital signs carts, shower chairs, shower beds, and patient lifts. The timing and location of each sampling event were recorded. 105 samples were collected, cultured on selective media (for methicillin-resistant Staphylococcus aureus (MRSA), vancomycin-resistant Enterococcus species, extended-spectrum ?-lactamase producing Enterobacteriaceae, and multidrug-resistant Acinetobacter species), and presumptive MDRO isolates were MALDI-TOF confirmed and whole genome sequenced. We assessed pairwise genomic relatedness using split-kmer analysis for any MDRO species that was isolated at least twice. We established genomic clusters based on two criteria: the proportion of kmers that matched between isolates (< 0.9) and a single-nucleotide polymorphism (SNP) threshold for each MDRO species based on established literature (e.g., < 7 SNPs). **Results:** In the 105 samples, four MDROs were detected more than once: vancomycin-resistant Enterococcus faecium (VREfm; N=17), methicillin-resistant Staphylococcus aureus (MRSA; N=9), Klebsiella pneumoniae (KP; N=4), and Acinetobacter baumannii (AB; N=2). To date, MRSA isolates have not been whole genome sequenced and are thus excluded from our results. MDRO detection rates varied across species and sample locations (Table 1). To assess whether these detections indicated pathogen movement, we assessed genomic relatedness (Figure 1) and found: 12 out of 17 VREfm isolates belonged to three multi-isolate clusters of sizes ranging from two to seven isolates; three out of four KP isolates formed a single cluster; while the two AB isolates were unrelated. All four clusters that were detected in the study included isolates collected from shared MME. Three of the four clusters detected involved samples taken in more than one location within the ward, with the largest cluster involving VRE isolates collected from five unique patient rooms and three equipment types stored in a hallway (Figure 1). **Conclusions:** Our findings suggest that MME may be an important reservoir for MDROs and can facilitate pathogen movement between patient rooms in SNFs. Adequate cleaning of shared MME is necessary to address the risk of fomite-based

**Figure:**
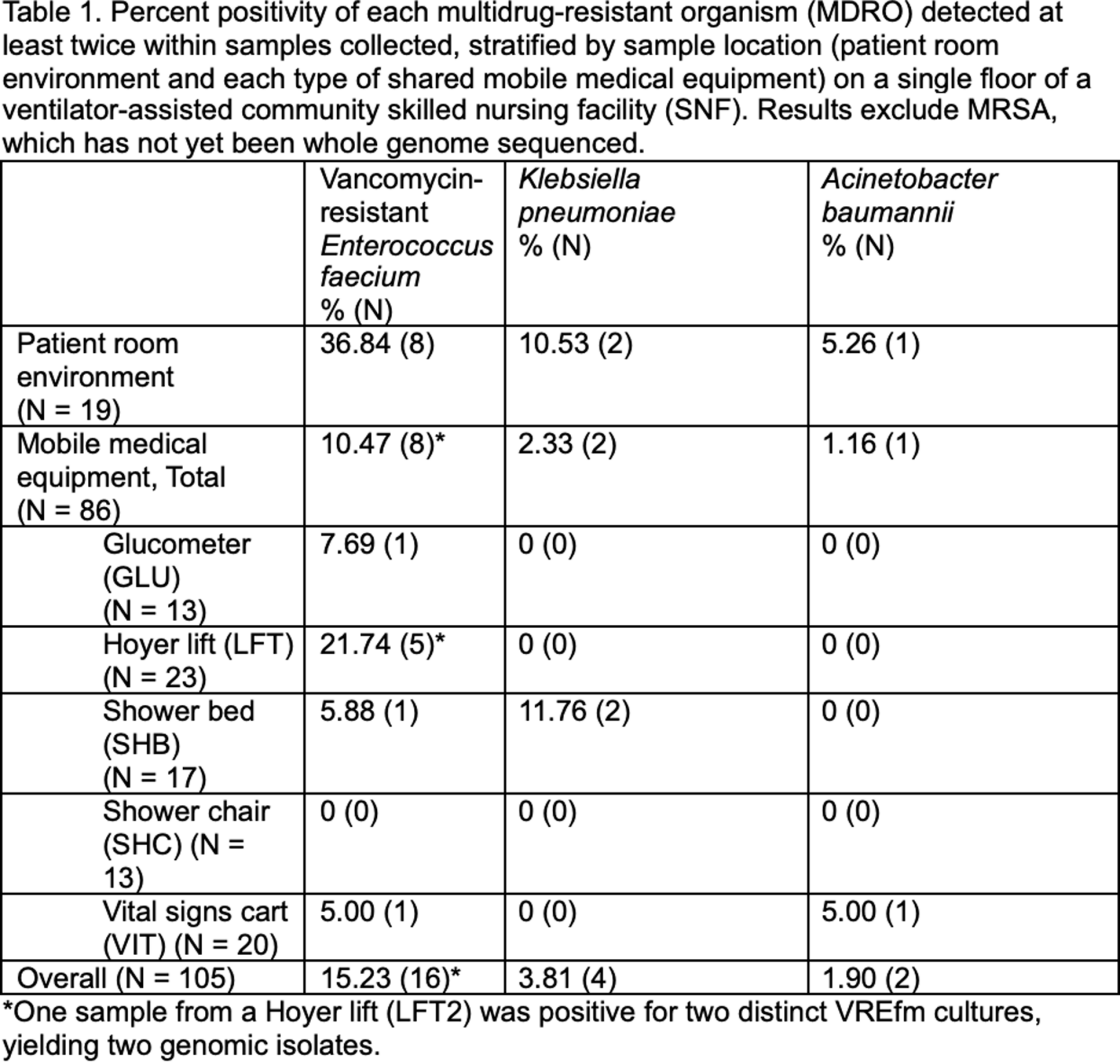

**Figure:**